# Standard Addition Quantitative Real-Time PCR (SAQPCR): A Novel Approach for Determination of Transgene Copy Number Avoiding PCR Efficiency Estimation

**DOI:** 10.1371/journal.pone.0053489

**Published:** 2013-01-07

**Authors:** Yuji Huang, Xueren Yin, Changqing Zhu, Weiwei Wang, Donald Grierson, Changjie Xu, Kunsong Chen

**Affiliations:** 1 Laboratory of Fruit Quality Biology/The State Agriculture Ministry Laboratory of Horticultural Plant Growth, Development and Quality Improvement, Zhejiang University, Hangzhou, P. R. China; 2 Plant and Crop Sciences Division, School of Biosciences, University of Nottingham, Sutton Bonington Campus, Loughborough, United Kingdom; TGen, United States of America

## Abstract

Quantitative real-time polymerase chain reaction (qPCR) has been previously applied to estimate transgene copy number in transgenic plants. However, the results can be erroneous owing to inaccurate estimation of PCR efficiency. Here, a novel qPCR approach, named standard addition qPCR (SAQPCR), was devised to accurately determine transgene copy number without the necessity of obtaining PCR efficiency data. The procedures and the mathematical basis for the approach are described. A recombinant plasmid harboring both the internal reference gene and the integrated target gene was constructed to serve as the standard DNA. It was found that addition of suitable amounts of standard DNA to test samples did not affect PCR efficiency, and the guidance for selection of suitable cycle numbers for analysis was established. Samples from six individual T_0_ tomato (*Solanum lycopersicum*) plants were analyzed by SAQPCR, and the results confirmed by Southern blot analysis. The approach produced accurate results and required only small amounts of plant tissue. It can be generally applied to analysis of different plants and transgenes. In addition, it can also be applied to zygosity analysis.

## Introduction

Plant genetic transformation is important for both basic plant biology research and industrial crop improvement. For example, through tomato transformation, Hamilton *et al*. [1], [2] proved that *pTOM13* encodes 1-aminocyclopropane-1-carboxylic acid oxidase, a key enzyme for ethylene biosynthesis; and Butelli *et al*. [3] observed that anthocyanin accumulation in tomato fruit requires coordinated regulation of MYB and basic helix-loop-helix (bHLH) transcription factors. Meanwhile, some successful genetically modified crops, including herbicide/pest resistant soybean, maize, cotton and oilseed rape, as well as nutritionally enriched golden rice, have already been commercially planted.

During plant genetic transformation, foreign DNA is randomly inserted into the plant genome as single or multiple copies. Frequently, selection of single-copy transgenic plants is a prerequisite for subsequent studies. This is often conducted through traditional approaches, such as Southern blot [1], [3], [4] and T-DNA flanking sequence analysis [5]. However, these techniques have their disadvantages. The Southern blot analysis requires relatively large quantities of DNA, and therefore a large amount of transgenic plant material, which is not available at the early seedling stage, while the T-DNA flanking sequence analysis is technically unstable and complicated owing to possible rearrangement or broken-end structure of integrated T-DNA [6].

Recently, quantitative real-time PCR (qPCR) has also been applied to estimate transgene copy number [7]–[16]. Unlike the traditional hybridization-based methods, qPCR assay allows more samples to be analyzed in a shorter time and requires much less plant tissue. However, despite its increasing application, there are controversies concerning its accuracy. In some studies, the results of transgene copy number determination have shown mismatches between qPCR and Southern blot analysis [8], [12], [13], [15], [17]. Some researchers have suggested that qPCR can only be viewed as complementary to Southern blot analysis for determination of transgene copy number, and that the technique is not sufficiently accurate and reproducible to discriminate twofold differences in transgene copy number [10], [17]. Thus, the accurate determination of transgene copy number by qPCR is not always possible by these approaches.

In a survey of previous literature on qPCR-based transgene copy number determination, it was found that some studies assumed that the PCR efficiency was 100%, or took for granted that efficiencies for the integrated target gene (*t*) and the internal reference gene (*r*) were equal, while most other studies estimated PCR efficiency either through the establishment of a standard curve by serial dilution [8], [13], or a mathematical calculation from the amplification curve itself [18]–[21]. However, none of these strategies is perfect. The true PCR efficiency cannot be 100%, and the efficiency for *t* and *r* can sometimes be quite different. PCR efficiency estimation based on a serial dilution standard curve can be erroneous because only one PCR efficiency value is generated, but this cannot hold true for serial samples where the concentrations of template DNA and potential inhibitors varies over several orders of magnitude [20], [22]–[25]. Also, estimation from the amplification curve itself can sometimes be quite difficult and complicated, and the results can vary between different approaches. Furthermore, even using the identical approach, the results can still be inconsistent when different analysis parameter settings for calculation were selected. In summary, it is often difficult to obtain accurate PCR efficiency, which results in possible erroneous estimation of transgene copy number from qPCR.

In this study, we present a novel qPCR approach, named standard addition qPCR (SAQPCR), to accurately determine transgene copy number. The strategy is to add known amounts of standard DNA to test samples to change fluorescence intensity and Ct values, which is similar to standard addition in quantitative chemical analysis [26]. In this assay, the estimation of PCR efficiency can be bypassed, which is not the case in the previously mentioned approaches.

## Materials and Methods

### Theoretical Basis for Determination of Transgene Copy Number by SAQPCR

The fluorescence produced during the qPCR exponential amplification phase is dependent on several factors as indicated in the following equation:
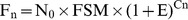
(1)Where F_n_ is fluorescence intensity; N_0_ is initial number of molecules of the investigated gene; FSM is the fluorescence of a single DNA molecule of a specific size, such as that of the PCR product; E is PCR efficiency; and C_n_ indicates cycle number.

**Figure 1:**
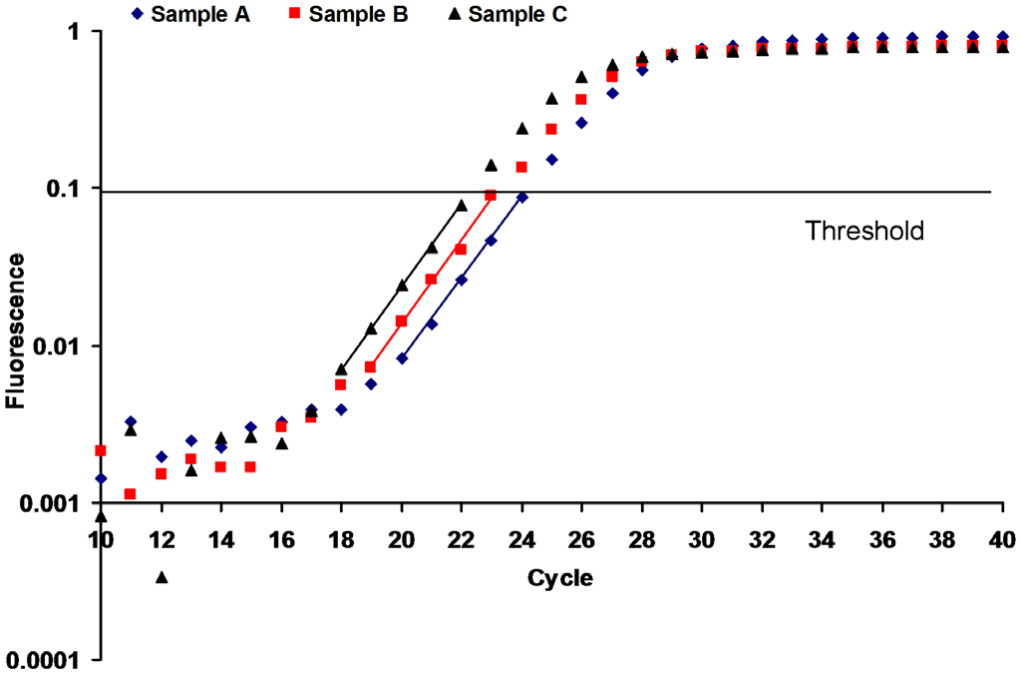
Effects of standard DNA addition on fluorescence intensity and C_t_. Oblique lines: exponential amplification phases suggested by LinRegPCR; Sample A: with 1 µl of tomato genomic DNA (10.20 ng µl^−1^, containing 10,000 *ELIP* molecules µl^−1^) as PCR template; Sample B: with 1 µl tomato genomic DNA plus 1 µl of pHE (0.051 pg µl^−1^, containing 10,000 *ELIP* molecules µl^−1^) as PCR template; Sample C: with 1 µl tomato genomic DNA plus 3 µl of pHE as PCR template.

Then for the internal reference gene (*r*) and the integrated target gene (*t*), the following equations apply:
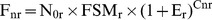
(2)

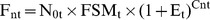
(3)Since the PCR product size for *r* and *t* is same in this approach, and the fluorescence intensity was recorded under the same conditions, FSM_r_ can be regarded as equal to FSM_t_. Therefore, the following equation can be obtained from Equation (2) divided by the Equation (3).
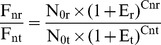
(4)And,



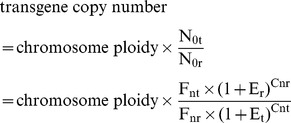
(5)For selected values of C_nt_ and C_nr_, F_r_ and F_t_ data can be obtained, and therefore, in the above equation E is the only unknown parameter required for copy number determination. However, as described above in Introduction Section, PCR efficiency cannot be accurately determined either based on a serial dilution standard curve or from the amplification curve itself. Similar situations also occur in quantitative chemical analysis, where accurate determination of analytes in test samples is often interfered with by impurities present, and an approach named ‘standard addition’ is frequently used to solve this matrix effect problem [25]. In this study, a similar strategy was applied to avoid the necessity of estimating PCR efficiency. Different known amounts (0, S, 3S, where S is equal to the estimated N_0_ of *r*, which was set at 10,000 molecules in this study) of standard DNA, the recombinant plasmid pHE in this study, were added to test samples. For the qPCR of samples following standard addition of different amounts, the following three equations can be obtained.
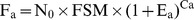
(6)

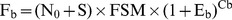
(7)

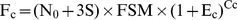
(8)Where C_a_, C_b_ and C_c_ indicate cycles within the exponential amplification phase (Figure 1).

Assuming that the addition of suitable amounts of standard DNA to the test samples does not significantly affect PCR efficiency (which was confirmed as described later in Results and Discussion Section), the PCR efficiencies both before and after standard DNA addition were set as E, and Equations (6)–(8) can be re-written as:
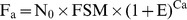
(9)

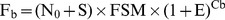
(10)

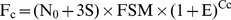
(11)Following the addition of standard DNA, a sequence where C_ta_>C_tb_>C_tc_ was produced (Figure 1). Setting I_b_ as the integer part for C_tb_ (the reason is given below), and C_a_ = I_b_ +1, C_b_ = I_b_ as well as C_c_ = I_b_ − 1, Equations (9)–(11) can be revised as follows:
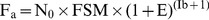
(12)

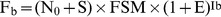
(13)

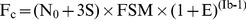
(14)


Therefore, the ratios of F_a_ to F_b_ and F_b_ to F_c_ can be calculated as follows:



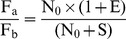
(15)

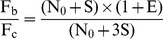
(16)Then the following equation can be obtained from Equation (15) divided by Equation (16).
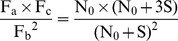
(17)To simplify the formula, set R = (Fa×Fc)/Fb2, then the following equation can be derived.
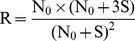
(18)In Equation (18), N0 is the only unknown factor and can be calculated as follows:
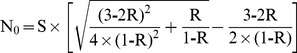
(19)A Microsoft Excel program (Program S1) was designed to facilitate calculation of N0.

N_0_ of *r* and *t* was determined respectively. As the addition amount S for both *r* and *t* were set equal to the estimated N_0_ of *r*. Therefore, S_r_ is equal to S_t_, and consequently,
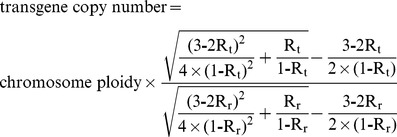
(20)


### Transgenic Tomato Plants

Transgenic tomato (*Solanum lycopersicum* Linn. cv. Ailsa Craig) plants, carrying an integrated *Escherichia coli* hygromycin phosphotransferase gene (*HPT*), were previously obtained *via Agrobacterium*-mediated transformation. Primary transformants (T_0_) lines and wild type plants were used in this study.

### Genomic DNA Extraction

Young leaves were collected and ground to a fine powder in liquid nitrogen. Approximately 100 mg of leaf tissue was used to isolate DNA for PCR with a DNeasy Plant Mini kit (Qiagen), according to the manufacturer’s instructions. Approximately 1 g of leaf tissue was used to isolate DNA for Southern blot following an improved CTAB method [27].

### Primer Design

Tomato early light-induced protein (*ELIP*) gene, a single-copy gene [28] was selected as the internal reference gene (*r*), and *E. coli HPT* as the integrated target gene (*t*) in this study. Primer pairs were designed for gene cloning or qPCR, using Primer Premier 5 (PREMIER Biosoft International) according to sequences deposited in GenBank (Table 1).

**Table 1:** Primers used in this study.

Gene	GenBank ID	Forward primer (5′–3′)	Reverse primer (5′–3′)	Application	PCR product size (bp)
*ELIP*	AY547273	GGTTCGCGATCTAGACAATACT^a^	CAAAATGAAAAGCTTTATATACTC^b^	cloning	1019
		ACAATACTAGTACTTCTTCACCTTT	AACACGCGAAGTCCTATGAA	qPCR	200
*HPT*	V01499	GCCTGAACTCACCGCGACGTCTG	CAGCACTCGTCCGAGGGCAAAGG	cloning	1013
		GCTCCGCATTGGTCTTGA	GGCGTCGGTTTCCACTAT	qPCR	200
		GATCGTTATGTTTATCGGCACT	TTGGCGACCTCGTATTGG	probe labeling	515

a The underlined nucleotides are the restriction site for *Xba* I; ^b^ The underlined nucleotides are the restriction site for *Hind* III.

### Recombinant Plasmid Construction

**Figure 2:**
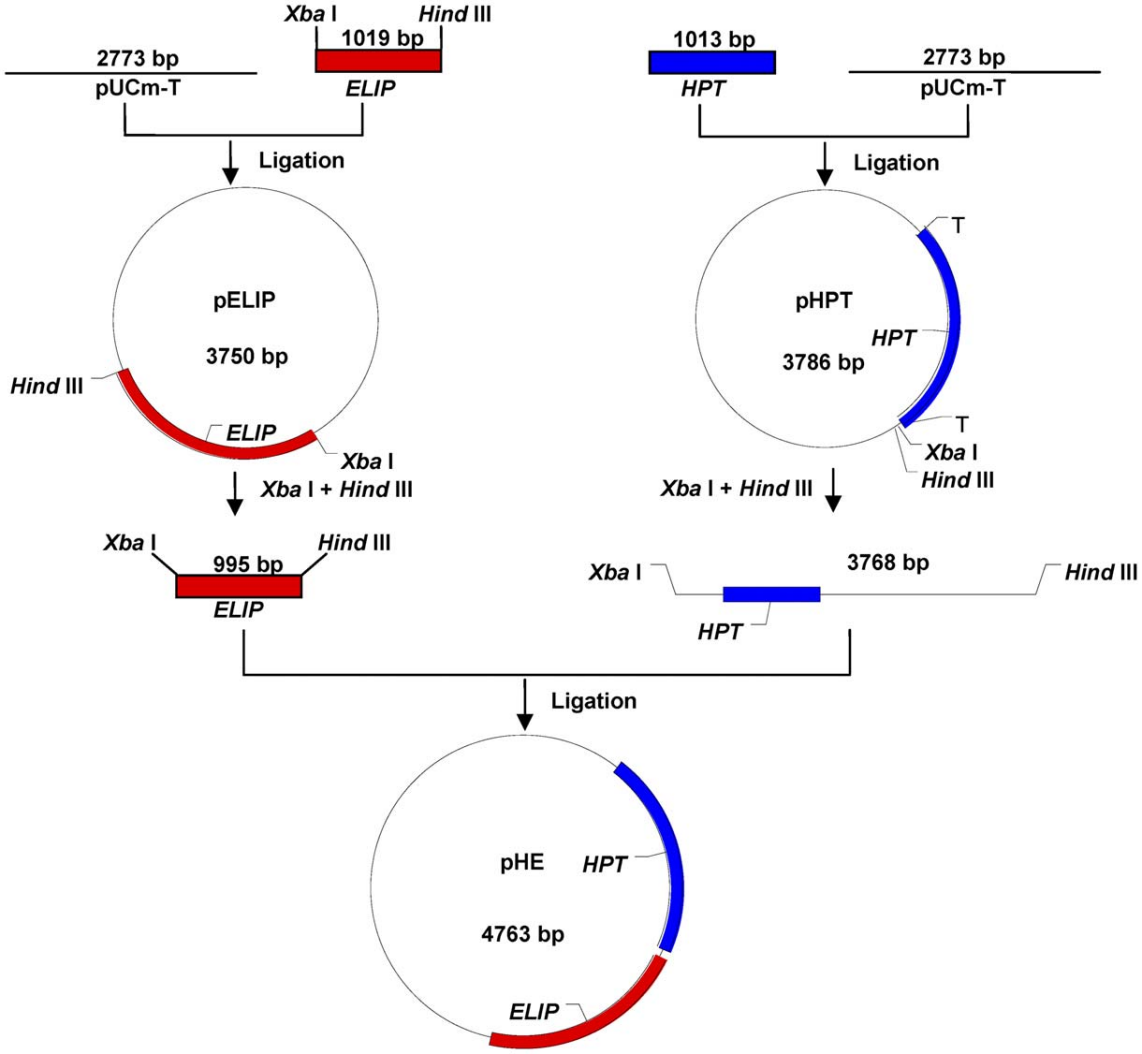
Construction of recombinant plasmid pHE. pELIP: plasmid harboring tomato *ELIP* gene; pHPT: plasmid harboring *Escherichia coli HPT* gene; pHE: plasmid harboring both tomato *ELIP* and *E. coli HPT* gene.

*ELIP* and *HPT* were amplified by PCR and then ligated into pUCm-T (TaKaRa) to generate plasmids pELIP and pHPT according to traditional protocols of Sambrook and Russell [29]. Following simultaneous digestion of the plasmids with *Xba* I and *Hind* III, the 995-bp fragment from pELIP digestion and the 3768-bp fragment from pHPT digestion were recovered and ligated to generate recombinant plasmid pHE (Figure 2). The authenticity of the recombinant plasmids was confirmed by sequencing.

### DNA Quantification

The tomato genomic DNA and pHE were quantified fluorescently using a Quant-iT™ PicoGreen® dsDNA Assay Kit (Invitrogen) and a Nano Drop 3300 Fluorospectrometer (Thermo Scientific).

### qPCR

qPCR reactions were performed in a LightCycler® (Roche) in a final volume of 12.50 µl, including 6.25 µl of SYBR® Premix Ex Taq™ (Takara), 0.25 µl of each primer (10 µM), 1 µl of tomato genomic DNA (10.20 ng µl^−1^), different amounts (0, 1 or 3 µl) of pHE (0.051 pg µl^−1^) (Table 2), made up to volume with PCR-grade water. The amplification program was initiated with a preliminary step of 30 s at 95°C, followed by 45 cycles of 5 s at 95°C, 20 s at 60°C, and 20 s at 72°C. A melting curve was generated for each sample at the end of each run to ensure the purity of the amplified products. qPCR was performed with five replicates.

**Table 2:** Effects of pHE DNA addition on PCR efficiency.

Sample^a^	Volume of tomato genomic DNA (µl)^b^	Volume of pHE (µl)^c^	*ELIP*			*HPT*		
			Average	5% level	1% level	Average	5% level	1% level
A	1	0	1.887±0.036	a	A	1.876±0.041	a	A
B	1	1	1.875±0.026	a	A	1.839±0.018	a	A
C	1	3	1.865±0.027	a	A	1.880±0.024	a	A

a A two-copy transgenic T_0_ tomato plant was used;

b The concentration was 10.20 ng µl^−1^, containing 10,000 *ELIP* and 10,000 *HPT* molecules µl^−1^;

c The concentration was 0.051 pg µl^−1^, containing 10,000 *ELIP* and 10,000 *HPT* molecules µl^−1^.

### PCR Efficiency Estimation

PCR efficiencies were estimated based on amplification curves using LinRegPCR [21]. The fluorescence threshold was recorded by the affiliated software of Roche LightCycler 1.5.

### Southern Blot

Digoxigenin (DIG)-based Southern blot was performed. 15 µg of tomato genomic DNA was digested with *Hind* III and *Bam*H I, respectively. Only one restriction site for these two restriction enzymes exists in the plant expression vector used for transformation and no site was present in the hybridization probe. The digested DNA was separated on 0.8% agarose gel, and then transferred to a positively charged nylon membrane (Roche) according to Sambrook and Russell [29]. DIG-labelled *HPT* probe DNA was prepared by PCR with the primers listed (Table 1), hybridization and autoradiography were performed according to DIG Application Manual for Filter Hybridization (Roche).

## Results and Discussion

### Addition of Suitable Amounts of Standard DNA to Test Samples Did Not Affect PCR Efficiency

Although PCR efficiency estimated from the amplification curve itself can be inaccurate and varies between different approaches and different analysis parameter settings, it can be reliably used to evaluate whether differences in the PCR efficiency exist between test samples when identical approach and analysis parameter settings were followed. In this study, PCR efficiencies were estimated with LinRegPCR [21] and the effects of standard DNA addition on changes in PCR efficiency were evaluated. With DNA from all six tomato plants analyzed, the addition of suitable amounts of standard DNA to the test samples did not affect PCR efficiency - see data from a two-copy T_0_ plant (Table 2). Therefore, the PCR efficiency of three samples, E_a_, E_b_ and E_c_ in Equations (6)–(8), can be replaced with the same value (E) in Equations (9)–(11) (Suggested above in Material and Methods Section).

### Guidance for Selection of Suitable Cycle Numbers for Analysis

**Figure 3:**
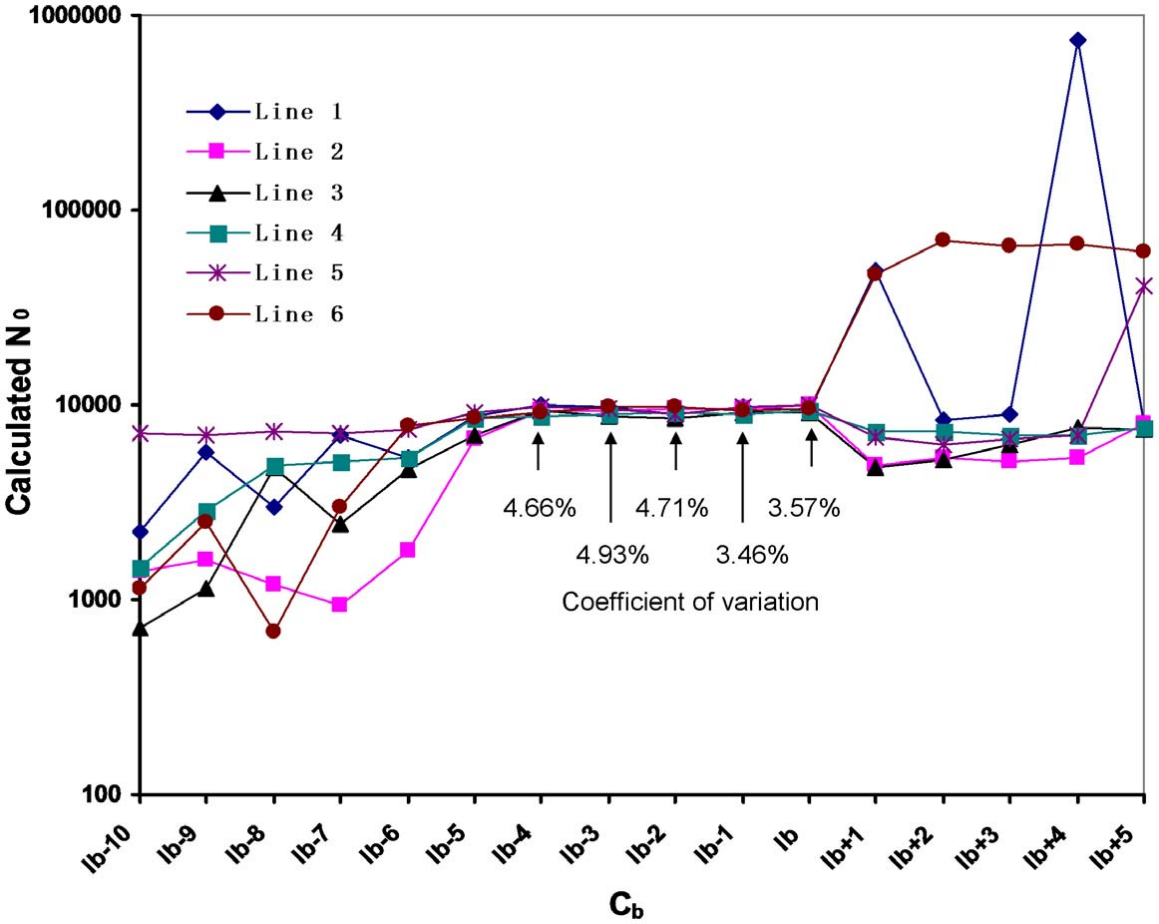
Calculation of N_0ELIP_ when taking a C_b_ within I_b_ −10 to I_b_ +5. The values above/beneath the arrows indicate the coefficient of variation. Line 1-Line 6 (L1-L6) were the six transgenic T_0_ tomato lines; I_b_ was set as the integer part for C_tb_; C_b_ indicated cycles in the exponential amplification phase of sample B.

Appropriate values of C_a_, C_b_ and C_c_ need to be selected. If too large, the cycles will not be in the exponential amplification phase; if too small, F_a_, F_b_, and F_c_ values will be correspondingly small and inaccurate because of the sensitivity of fluorescence detection. Therefore, using six transgenic tomato plants, *ELIP* N_0_ was calculated and compared for a value of C_b_ within I_b_ −10 to I_b_ +5. For C_b_ within I_b_ −4 to I_b_, the calculated *ELIP* N_0_ for each of the six analyzed plants was approximately 10,000 molecules (Figure 3), which was consistent with the estimated data from fluorospectrometric analysis. Moreover, since the six transgenic lines can be taken as six replicates for the calculation of *ELIP* N_0_, the coefficient of variation was used to evaluate the reproducibility of the data; the variance was <5% when C_b_ was within I_b_ −4 to I_b_. For simplicity, C_b_ = I_b_ was used in the present study.

### The Amount of Added pHE, S, Needs Not to Be Highly Accurate

N_0_ of the internal reference gene was estimated through fluorospectrometric quantification. This is highly recommended as the quantification obtained from another commonly used technique, UV spectrometric analysis, is not accurate due to severe interference from impurities commonly present in plant DNA samples. Sometimes, even with a fluorescence-based spectrometric technique, the quantification result may also not be highly accurate because of interference from impurities. This can also be the case for the standard DNA added.

However, with a recombinant plasmid harboring both *r* and *t* served as the standard DNA, the amount added to the test samples does not need to be highly accurate. As clearly shown in Equation (20), the transgene copy number result was independent of S, the amount of added standard DNA.

### Transgene Copy Number Determination of Six Transgenic Tomato Plants

The *HPT* gene copy numbers in transgenic tomato plants were determined by the ratio of N_0HPT_ to N_0ELIP_. The transgene copy number should be twice the ratio because of the diploid nature of tomato. In the present study, samples from six individual T_0_ plants were analyzed: the data indicated that three lines had one copy of the transgene, while the other three lines had two copies (Table 3).

**Table 3:** Determination of transgene copy number of six transgenic tomato plants by standard addition qPCR (SAQPCR).

Line	N_0HPT_	N_0ELIP_	N_0HPT_/N_0ELIP_	Transgene copy number^a^
1	4476.61	9273.31	0.48	1
2	4719.62	9912.54	0.48	1
3	4699.55	9084.33	0.51	1
4	9386.66	9361.90	1.00	2
5	9672.54	9901.20	0.97	2
6	9635.41	9565.91	1.01	2

A quantified amount of 10.20 ng of tomato genomic DNA, which contains around 10,000 molecules of *ELIP*, was included in each PCR reaction.

a Refers to the copy number per diploid genome.

**Figure 4:**
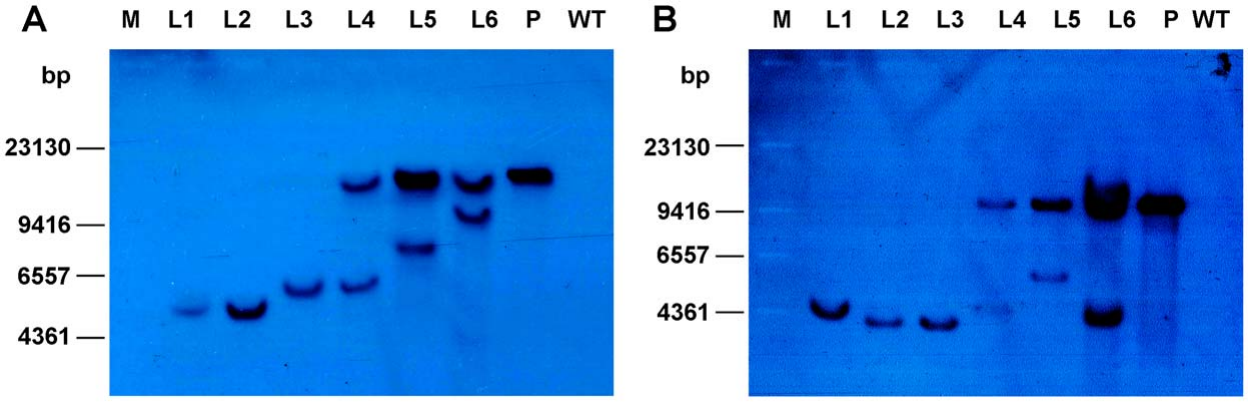
Southern blot analysis of six transgenic tomato T_0_ lines. A) *Hind* III digestion; B) *BamH* I digestion. M, λDNA/*Hind* III marker; L1-L6, transgenic tomato T_0_ lines, were the same as those appeared in Figure 3 and Table 3; P, positive control (plasmid); WT, negative control (wild type).

The transgene copy number of these six transgenic tomato plants was also analyzed by Southern blot (Figure 4), which confirmed the SAQPCR results obtained in the present study.

### Prospects for the Application of SAQPCR Approach to other Plants and Organisms

The transgene copy number of six transgenic tomato plants obtained by the SAQPCR approach was confirmed by Southern blot analysis (compare results in Table 3 and Figure 4). However, unlike the latter, only a small amount of plant tissue is required for SAQPCR, and the analysis can be completed in a much shorter time. Therefore, the approach is especially suitable for high throughput transgene copy number determination in small-sized plant species or cultivars, as well as slow-growing woody plants.

Although the approach was established on tomato with single-copy *ELIP* as the *r* and *HPT* as *t*, it is generally suitable for other plants and genes. Using transgenic kumquat (*Fortunella crassifolia* Swingle) [4], a type of citrus, with the single-copy mitochondrial citrate synthase gene (GenBank U19481) [30] serving as the *r* and the *Escherichia coli* neomycin phosphotransferase gene (*NPT II*) (GenBank V00618) as the *t*, we applied the protocol to quantify the transgene copy number of transgenic plants B2, C6 and C9, with results suggesting the former two lines are single copy per diploid genome and the third having three copies, which is consistent with the conclusions obtained from Southern blot analysis previously [4]. This approach should be also suitable for transgene copy number determination in other organisms although this has not been tested in this study. Furthermore, this approach can also readily be adapted for zygosity analysis of filial transgenic organisms.

### Summarized General SAQPCR and Data Analysis Protocol for Transgene Copy Number Determination

Based on the procedures established in the present study, a general SAQPCR and data analysis protocol is summarized as following.

1. Select a single-copy internal reference gene (r) and an integrated target gene (t).
2. Construct the recombinant plasmid (pRT) harboring both r and t. After fluorescence quantification, dilute pRT to S (10,000 was recommended) molecules per microliter.
3. Extract genomic DNA of previously identified transgenic plants, and after fluorescence quantification, dilute to the concentration containing S molecules of r per microliter.
4. Set up template DNA series for qPCR analysis. Samples A, B, and C all contain 1µl of diluted plant genomic DNA, while they contain 0, 1µl and 3µl of diluted pRT DNA, respectively.
5. Perform qPCR with five replicates.
6. Obtain amplification data (fluorescence vs. cycle) as well as Ct.
7. Set I_b_ as the integer part for C_tb_, and C_a_ = I_b_+1, C_b_ = I_b_ as well as C_c_ = I_b_−1. Obtain the corresponding fluorescence intensity data, F_a_, F_b_, F_c_.
8. Calculate R_r_ and then N_0r_ according to following equations.

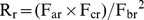


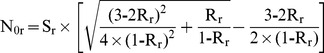



Note: a Microsoft Excel program (Program S1) was designed to facilitate calculation of N_0_.

1. Similarly, calculate R_t_ and then N_0t_ according to following equations.

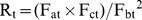


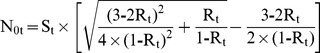

1. Calculate transgene copy number according to following equation.

Transgene copy number = chromosome ploidy×N_0t_/N_0r_.

### Conclusions

A novel approach, SAQPCR, was established in the present study to accurately determine the transgene copy number without the necessity of obtaining PCR efficiency data. The strategy is to add a known amount of standard DNA, a recombinant plasmid harboring both *r* and *t*, to the test samples to change fluorescence intensity and C_t_ values. On the basis of correlation between fluorescence value and the added DNA amount, a mathematical equation was reached to calculate the transgene copy number without the necessity of obtaining PCR efficiency data.

## Supporting Information

Program S1 **A Microsoft Excel program for starting amount (N_0_) calculation by standard addition real-time PCR (SAQPCR).**
(XLS)
